# Open-access clinical trial registries: the Italian scenario

**DOI:** 10.1186/1745-6215-13-194

**Published:** 2012-10-18

**Authors:** Paola Mosconi, Anna Roberto

**Affiliations:** 1 Laboratory of Medical Research and Consumer Involvement, Mario Negri Institute for Pharmacological Research, Via La Masa 19, 20156, Milan, Italy

**Keywords:** Clinical trials registry, Citizen, Information

## Abstract

**Background:**

Citizens, patients and their representatives are increasingly insisting on working with health professionals to organize and discuss research protocols. The International Committee of Medical Journal Editors recommended setting up a public clinical trial registry where anyone can find key information about a trial. Around the world, governments have, in fact, now begun to legislate mandatory disclosure of all clinical trials. The aims of the present survey were to assess the availability of clinical trial registries for Italian citizens and to examine the transparency of the data items reported.

**Methods:**

The availability of open-access clinical trial registries was surveyed on a sample of 182 websites, including research institutes and centers of excellence (IRCCS-teaching hospitals), hospitals and associations. For each registry we downloaded a sample of two trials to assess the correspondence of the data items reported. Results from the Italian and international registries were compared.

**Results:**

Fifteen percent of the sample had an open-access registry of clinical trials. Comparison of the data items available, in terms of completeness and transparency, from institutional and international registries indicated wide variability.

**Conclusions:**

Italian citizens, patients and their associations have scant access to local registries of clinical trials, and international registries are generally more informative. On the European level, advocacy and lobby actions are needed among citizens and patients to boost the diffusion of open-access clinical trial registries without language barriers, thereby facilitating participation, access to information, and the coordination of clinical research.

## Background

Demand from citizens, patients and their representatives for information about clinical trials (CT) has been growing for many years [1,2]. Laypeople are increasingly aware of the need to participate in health decisions and want a more active role rather than just that of a passive subject in a CT [3,4]. Published personal and collective experiences very clearly report that patients’ needs are unmet in the research agenda [5-7]. These unmet needs are related to several aspects such as the choice of primary endpoints for CT relevant for patients, the increase in independent head-to-head comparison studies of different pharmacological or assistance strategies, the access to easy-to-understand lay-language information about on-going CT, the rapid publication of all results whether positive or negative, and, last but not least, a research agenda designed according to the priorities discussed by all the stakeholders, including citizen and patients or their representatives.

A recent review states that making the patient more aware makes a CT more acceptable and increases the number of subjects recruited, but the persisting lack of information does not help patients decide whether and how to participate in clinical research [8]. Citizens’ and patients’ organizations are seeking to set up working groups with clinical researchers through training programs [9], discussion of research protocols [10,11], or information projects. Investigators are being urged to spread more information on ongoing CT and results [4].

Currently in industrialized countries approval to start a CT must be obtained from ethics committees, and informed consent must be obtained from trial participants. According to the Declaration of Helsinki, patients who take part in a CT must be adequately informed on the aims, methods, expected benefits and potential risks of participation, and the latest version specifies that before recruitment of the first subject every CT must be registered in an open-access registry [12].

Despite a moral obligation to report the positive or negative results of any research on humans, some sponsors keep quiet about negative findings when publishing their data, or when submitting them to regulatory authorities [13]. This also happens with CTs involving participants in low- and middle-income countries with inadequate oversight mechanisms [14].

The International Committee of Medical Journal Editors (ICMJE) has promoted the establishment of a public clinical trials registry (CTR) [15]. Its purpose would be to promote access to information so everyone can find key information about CTs, and also to help shape medical decision-making. Since that decision, many governments have made disclosure of all CTs mandatory [16]. Since 2004 the transparency of CTs involving children in four European countries (France, Italy, United Kingdom and Spain) has been guaranteed by the DEC-net registry (Drug Evaluation in Children - the European registry of CTs on medicines for children). In 2004 DEC-net was one of only three international records compliant with the ICMJE and World Health Organization (WHO) rules. DEC-net’s work monitoring ongoing studies means it can potentially help avoid trial duplication and facilitate better distribution of funds based on the unmet therapeutic needs of children [17].

Since March 2011, after years of use restricted to national authorities, all European citizens able to read English have had free access to information on drug CTs approved in Europe, thanks to the new registry created by the European Medicines Agency (EMA) [18]. The registry was assembled after extensive consultations with all those concerned, such as patients and health professionals, to ensure that their needs were taken into account during its design [19].

In Italy, the national registry on CTs of medicines has been active since 2000, developed by the Italian Medicines Agency (AIFA), a national public body responsible for drug regulation operating under the direction of the Ministry of Health, with access limited to health professionals. In 2005, this registry of CTs on drug comparison was made accessible to citizens as well.

To evaluate the availability of open-access CTRs for citizens and patients and to evaluate the transparency of the data items in comparison with international registries, we carried out this survey in several hospitals, research centers and association websites.

## Methods

We visited a sample of 182 official Italian websites between March 7 and April 20 2011:

 • All IRCCS-teaching hospitals, that is, 42 research institutes and centers of excellence,

 • A convenience sample of public hospitals involved in CT multicenter projects: CERP (collaborative group for cancer pain, n=59), MANGO and MITO (collaborative groups for ovarian cancer, n=65) [20,21],

 • A convenience sample of 16 national associations^1^ or federations of patients involved in the Mario Negri Institute PartecipaSalute project [22].

We checked all the websites for open-access CTR, or for a section on CTs in progress, or at least for a link to other registries.

### Data items collected

For each CTR identified we recorded whether the following data items were collected: unique trial number, title, pathology, presence or absence of treatment, type or phase of the study, approval by the ethics committee, sponsor, aim, inclusion and exclusion criteria, primary and secondary outcomes, starting date, number of patients needed, recruiting status, contact persons, language of the registry, presence of a glossary, updating of the website/registry, considering December 2010 as closing date, and any publication or references related to the CTs. These are recognized as the 20 minimum items to be collected in a registry, as required by the World Health Organization [23].

For each Italian CTR found we checked the presence or absence of the data items, and to verify how the data items list corresponded two CTs were randomly downloaded and printed. Similarly, two CTs were downloaded from CTRs of the 11 international institutions^2^ from the International Clinical Trials Registry Platform **(**ICTRP) database [24], and of AIFA, where only drug CTs are reported. Descriptive statistics, mainly proportions, were used to analyze all data collected.

## Results

Out of 182 Italian health institution websites visited, 15 (8%) had CTR for consultation by citizens, nine in the north of Italy, five in the center and one national association (Table 1). None of those without a CTR, had a link to another registry or the AIFA or European registry. One IRCCS-teaching hospital mentioned that it was possible to participate in the trial after an appointment with an oncologist.

**Table 1:** Registries of clinical trials in the institutions surveyed

	**Number**	**YES (%)**	**NO (%)**
IRCCS-teaching hospitals	42	11 (26)	31 (74)
Hospitals	124	3 (2)	121 (98)
Associations	16	1 (6)	15 (94)
**TOTAL**	182	15 (8)	167 (92)

We compared the availability of data items on the CTs reported in Italian CTRs with international registries (Table 2). The results varied widely; the AIFA registry has all data items reported in each CT’s record.

**Table 2:** Data items collected in databases supporting the clinical trial registry

	**IRCCS, hospitals, associations**	**AIFA**	**International institution**
**Data items**	**(15)**	**(1)**	**(11)**
	%	%	%
Unique trial number	13	100	100
Title	80	100	100
Pathology	93	100	100
Treatment	60	100	100
Type/phase	67	100	100
Ethics committee	20	100	55
Primary sponsor	67	-	100
Aim of the study	47	100	100
Inclusion criteria	7	100	100
Exclusion criteria	7	100	100
Primary endpoint	13	100	100
Secondary endpoint	13	100	100
Start of trial	7	100	100
Sample size	20	100	100
Recruitment status	33	100	100
Contact	67	-	100
Language			
Italian	80	100	-
Italian +English	20	-	100
Glossary	7	-	-
Web update	93	100	91
Publication*	13	-	27

*Related to the CTs.

Generally, the registries almost always collected the title and pathology in the database (80-93%), the trial phase, sponsors and contacts (67%), type of treatment (60%) and aim of the trial (47%). Important data items such as inclusion and exclusion criteria or starting date were reported in only 7% of the Italian registries. All these percentages were higher in the international registries, many of them institutional. A glossary was very rare.

Because of the small number of CTR, it was impossible to compare IRCCS-teaching hospitals and public hospitals or associations.

## Discussion

The internet is a very important tool for retrieving health information [25], and increasing numbers of citizens use the web as an easy way to find information. Italian citizens and patients, however, have scant access to clinical information about CTs. The institutional AIFA registry, where only drug CTs are reported, is an open-access resource, but because it is not linked to hospitals or associations, patients and citizens are not aware of the existence of this useful information. In addition, the data items collected by the 15 Italian CTRs in our sample are not satisfactory, and are poor compared to international or AIFA CTRs. The ICMJE recommends a minimum set of data items for CTR [16]; our sample had from 7% to 93%. Considering the increasing interest of citizens and patients in participating in the debate on health and clinical research [5-7], it is important to have information on CTs that is easily accessible enabling people to ask for admission to a trial (for example, inclusion/exclusion criteria, telephone contact). It is also important to use non-technical language or at least to provide a glossary. The language used by Italian CTRs is mostly technical, and in only one case was ‘lay’ language used.

This study has some limits. First, it considered all the IRCCS-teaching hospitals, but only a convenience sample of associations or federations and public hospitals. However, all the hospitals were involved in CTs, and the associations/federations cover representative pathologies like cancer, cardiovascular disease and neurological disorders. Second, the international comparison only looked at the 11 registries set up by international institutions. Finally, the data refer only to the Italian scenario but demand for open-access, user-friendly, complete CTRs is worldwide [26-29], and this is an important challenge to facilitate the access to and participation in CTs.

## Conclusions

Non-English speaking European citizens or patients, such as Italians, face a significant language barrier in consulting international databases in English. However, the importance of access to information on CTs is internationally recognized, as recently discussed in India [27], Germany [28,29], and Australia [26].

Our results suggest that advocacy and lobby actions at local and international levels are needed among clinicians, researchers, and citizens or patients, and their organizations, to boost sensitivity to this issue and to clinical research in general (‘you get better care where they do research’) [4]. It is important to demand that every hospital, clinical research center, or patients’ organization, set up their own CTRs on their own websites [30] and - even more importantly – to create links to institutional open-access CTRs, like the available drug-oriented CTRs.

The EMA should continue to work with stakeholders to improve the EU Clinical Trials Registry, improving the quality and completeness of data and ease of consultation. EMA could have a strong driving effect, publishing a higher-quality registry, and promoting initiatives for all European countries.

## Endnotes

^1^List of associations (focus of the association’s activities is in brackets): AIRC (cancer); TELETHON (neurological disorders); AI (Alzheimer); AICE (epilepsy); AIMA (Alzheimer); AISM (multiple sclerosis); ANLAIDS (AIDS); CO.NA.CUORE (cardiovascular diseases); EUROPA DONNA (breast cancer); EUROPA UOMO (prostate cancer); AIMAC or FAVO (cancer); LILA (AIDS); FEDERASMA (asthma); FISH (handicap); AISLA (amyotrophic lateral sclerosis); FAND (diabetes).

^2^List of International registry: CLINICALTRIALS.GOV; ANZCTR (Australian New Zealand Clinical Trials Registry); WHO (World Health Organization); ISRCTN REGISTER; CHICTR (Chinese Clinical Trial Registry); DRKS (Deutsches Register Klinischer Studien); CTRI (Clinical Trials Registry- India); IRCT (Iranian Registry of Clinical Trials); NTR (Netherlands Trial Register); SLCTR (Sri Lanka Clinical Trial Registry); EU.CLINICALTRIALSREGISTER.

## Abbreviations

AIFA: Italian Medicines Agency; CT: clinical trial; CTR: clinical trials registry; DEC-net: Drug Evaluation in Children - the European registry of CTs on medicines for children; EMA: European Medicines Agency; ICMJE: International Committee of Medical Journal Editors; ICTRP: International Clinical Trials Registry Platform; WHO: World Health Organization.

## Competing interests

The authors declare that they have no competing interests.

## Authors’ contributions

The authors contributed equally to this work.

## Authors’ information

Paola Mosconi, Biol Sci D, Head of the Laboratory for medical research and consumer involvement, Istituto di Ricerche Farmacologiche Mario Negri, via La Masa 19, 20156 Milan; e-mail mosconi@marionegri.it Anna Roberto, Biol Sci D, Fellowship in the Laboratory for medical research and consumer involvement, Istituto di Ricerche Farmacologiche Mario Negri, via La Masa 19, 20156 Milan; e-mail anna.roberto@marionegri.it
